# Supplementary material for: Chromothripsis during telomere crisis is independent of NHEJ, and consistent with a replicative origin
Source: Genome Res. 2019 May;29(5):737–49. doi: 10.1101/gr.240705.118 (PMC6499312; doi:10.1101/gr.240705.118)
Supplement: Supplemental Material [file supp_gr.240705.118_Supplemental_file_1.zip › contigs/annotated_contigs/DB53/contig.2.DB53_length_1037_mean_cov_7.80520732883.docx]

**DB53_length_1037_mean_cov_7.80520732883**

```
ATTTCTTCTAGATGTTCTAGTTTATTTGCATAGAGGTGTTTATAGTATTCTCTGATGGTAGTTTGTATTTCTATGGGATCCGTGTTGAT
 >chr4:72864678-72865167 - E=4e-276
ATCCCCTTTATCATTCTTTATTGCATCTATTTGATTTTTCTCTCTTCTTTATTAGTCTTGCTAGCGGTCTATCAATTTTATTGATCTTT

TCAAACAACCTGCTCCTGGATTCATTGATTTTTTGCAGAGTTTTTTGTGTCTCTATCTCCTTCAGTTCTGCTCTGATCTTAGTTATTTC

TTGCCTTCTGCTAGCTTTTGAATTTGTTTGCTCCTGCTTCTCTAGTTCTTTTAATTGTGATGGTAGGGTGTTGATTTTAGATCTTTCCT

GCTTTCTCTTGTGGGCATTTAGTGCCATAAATTTCCCTGTACACACTGCTTTAAATGTGTCCCAGAGATTCTGGTATGTTGTGTCTTTG

TTCTCACTGGTTTCAAAGAACATCTTTATTTCTGCCTTCATT|TC|TCAGCATTTGCTTGTCTGTAAAGGATTTTATTTCTCCTTCACT
                                           >chr4:72863219-72863721 - E=3e-226
TATGAAGCTTAGTTTGGCTGGATATGAAATTCTGGGTTGAAAATTCTTTTCTTTAAGAATGTTGAATATTGGCCCCCACTCTGTTCTAG

CTTGTAGGGTTTCTGCCGAGAGATCTGCTGTTAGTCTGATGGGCTTCCCTTTGTGGGTAACCCGACCTTTCTCTCTGGCTGCTCTTAAC

ATTTTTTCCTTTATTTCAACCTTGGTGAATCTGAAAATTATGTGTCTTTTGGTTGTTCTTCTTGAGGAGTATCTTTGTGGTGTTCTCTG

TATTTCCTGAATTTGAATGTTGGCCTACCTTACTAGTTGGGGAAGTTCTCCTGGATAATATCCTGAAGAGTGTTTTCCAACTTGGTTCC

GTTCTCCCCATCACTTTCAGGTAATCAATCAAACATAGATTTGGTCTTTTCACATAGTCCCATATTCCTCGGAGGCTTTGTTCATTTCT

ATTTACTCTT|CTTTTCTCTCTACTTGCCTTTTGGCTTTATTTTATTATATTGATCTTCCT
```
